# Quality assurance data for regional drip-and-ship strategies- gearing up the transfer process

**DOI:** 10.1186/s42466-021-00136-x

**Published:** 2021-08-02

**Authors:** Erendira G. Boss, Ferdinand O. Bohmann, Björn Misselwitz, Manfred Kaps, Tobias Neumann-Haefelin, Waltraud Pfeilschifter, Natalia Kurka

**Affiliations:** 1 Department of Neurology, University Hospital Frankfurt, Frankfurt, Germany; 2 Institute of Quality Assurance Hesse, Eschborn, Germany; 3 Department of Neurology, Justus-Liebig-University Giessen, Giessen, Germany; 4 Department of Neurology, Klinikum Fulda, Fulda, Germany

**Keywords:** Stroke, Thrombectomy, DIDO, Interhospital transfer, Drip-and-ship, Mothership

## Abstract

**Background:**

Stroke patients with large vessel occlusion (LVO) require endovascular therapy (EVT) provided by comprehensive stroke centers (CSC). One strategy to achieve fast stroke symptom ‘onset to treatment’ times (OTT) is the preclinical selection of patients with severe stroke for direct transport to CSC. Another is the optimization of interhospital transfer workflow. Our aim was to investigate the dynamics of the OTT of ‘drip-and-ship’ patients as well as the current ‘door-in-door-out’ time (DIDO) and its determinants at representative regional German stroke units.

**Methods:**

We determined the numbers of all EVT treatments, ‘drip-and-ship’ and ‘direct-to-center’ patients and their median OTT from the mandatory quality assurance registry of the federal state of Hesse, Germany (2012–2019). Additionally, we captured process time stamps from primary stroke centers (PSC) in a consecutive registry of patients referred for EVT in our regional stroke network over a 3 months period.

**Results:**

Along with an increase of the EVT rate, the proportion of drip-and-ship patients grew steadily from 19.4% in 2012 to 31.3% in 2019. The time discrepancy for the median OTT between ‘drip-and-ship’ and ‘direct-to-center’ patients continuously declined from 173 to 74 min. The largest share of the DIDO (median 92, IQR 69–110) is spent with the organization of EVT and consecutive patient transfer.

**Conclusions:**

‘Drip-and-ship’ patients are an important and growing proportion of stroke patients undergoing EVT. The discrepancy in OTT for EVT between ‘drip-and-ship’ and ‘direct-to-center’ patients has been reduced considerably. Further optimization of the DIDO primarily aiming at the processes after the detection of LVO is urgently needed to improve stroke patient care.

**Supplementary Information:**

The online version contains supplementary material available at 10.1186/s42466-021-00136-x.

## Background

Patients with stroke due to acute large vessel occlusion (LVO) have a chance of only 10% to experience successful vessel recanalization under IV thrombolysis (IVT) alone [1]. Since this can be increased to > 70% by endovascular therapy [2], doubling the odds of an independent living status, all patients with stroke due to LVO should be given access to this therapy [2]. Hence, patients who are first admitted to a primary stroke center (PSC) without the capacity to perform thrombectomy have to be transferred to a comprehensive stroke center (CSC) after the detection of LVO.

This group of ‘drip-and-ship’ patients have significantly longer onset-to-treatment times (OTT) compared to patients admitted directly to a CSC (‘direct-to-center’) [3, 4] associated with less favourable outcomes [4, 5]. Several prospective ongoing randomized trials are currently investigating the benefit of a preclinical selection for EVT-candidates and direct routing to the nearest CSC compared to the standard ‘drip- and-ship’ concept [6–8].

The RACECAT study randomized severe strokes into either referral to the next primary stroke center (and thereby a potential “drip-and-ship” arm in case of LVO detection) or referral to the next comprehensive stroke center (“direct to center” arm) and surprisingly showed comparable functional outcomes in both groups 90 days after stroke [9]. It should be emphasized that these results were achieved on the basis of highly selected patients and excellent process times at the PSCs reflected by an onset-to-groin discrepancy of less than 1 h between direct-to-center and drip-and-ship patients [9].

There is very few information on the time toll of the ‘drip-and-ship’ approach from non-selective quality control registries that cover all patients transferred for EVT. These ‘real-life’ data provide transparency concerning the developments and current state of integrated acute stroke care. The workflow processes at the PSC are often not as well studied and understood as in CSC and increasingly move to the fore in the effort of optimizing OTT. In a similar treatment paradigm in cardiology, the term DIDO (‘door-in-door-out-time’) has been coined for the diagnostic processes and swift rerouting at a primary hospital without a catheter laboratory. In this context, a DIDO < 30 min has been shown to be associated with a greater likelihood of favourable outcome [10].

We aimed to evaluate the evolution of the OTT of ‘drip-and-ship’ patients in comparison to ‘direct-to-center’ patients in a virtually population-based statewide quality assurance registry and to identify the most important hurdles/impediments faced by PSC stroke teams aiming at a swift patient transfer towards thrombectomy.

## Methods

**Fig. 1:**
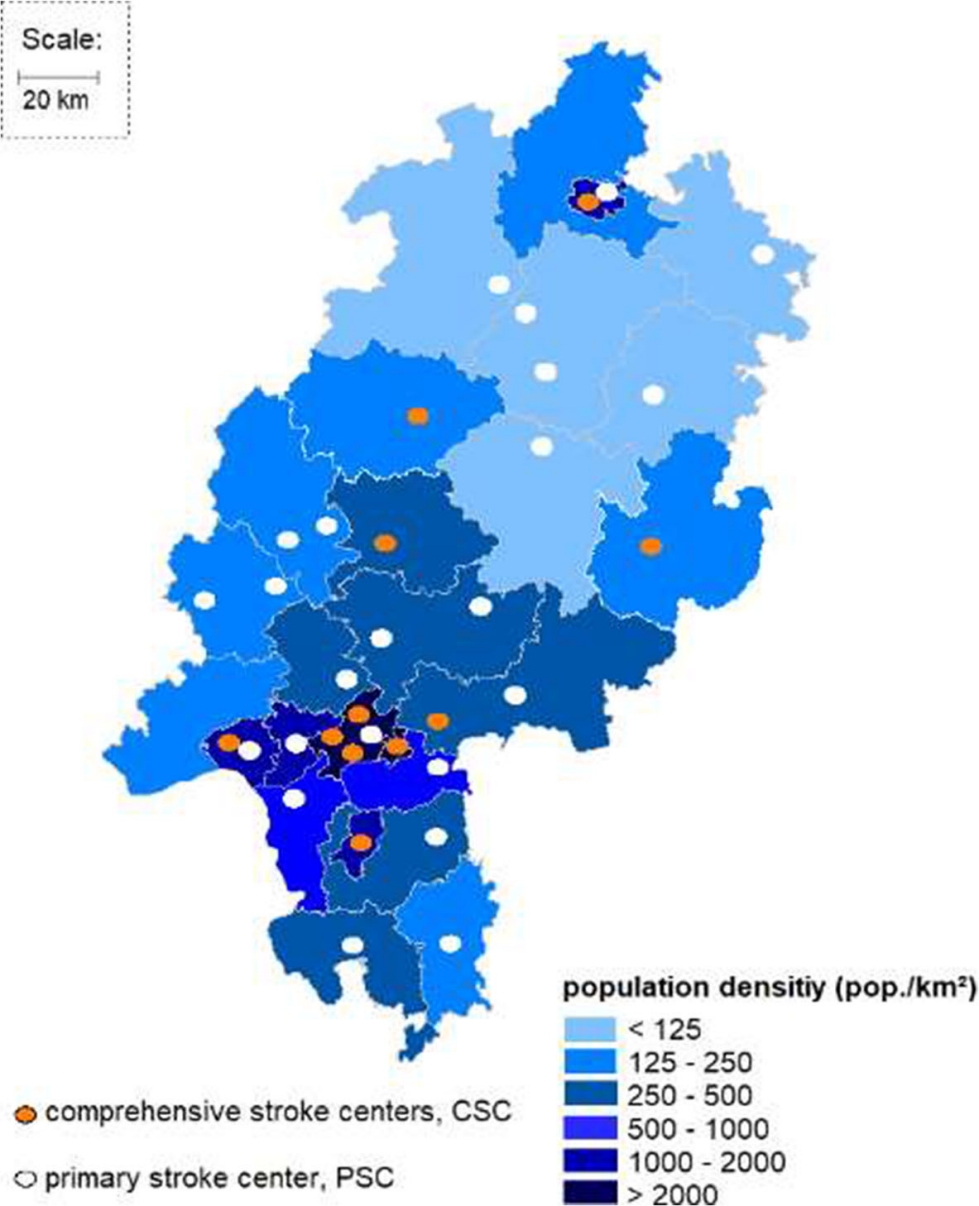
Primary stroke centers (PSC) and comprehensive stroke centers (CSC) in the federal state of Hesse. The federal state of Hesse (6.2 million inhabitants) and its population density depicted by district. The state is served by 11 CSCs and 32 PSCs

To analyze the evolution of the proportion among all EVT patients and the OTT dynamics of ‘drip-and-ship’ patients in comparison to ‘direct-to-center’ patients, we used full datasets of the federal quality assurance database for the state of Hesse, Germany [11, 12]. Data entry is mandatory for all inpatients with a final diagnosis of ischemic stroke (I63.x), transient ischemic attack (G45.x), intracerebral hemorrhage (I61.x) and subarachnoid hemorrhage (I60.x) yielding a virtually population-based dataset. The federal state of Hesse has 6.2 million inhabitants served by 11 CSCs and 32 PSCs (Fig. 1).

**Fig. 2:**
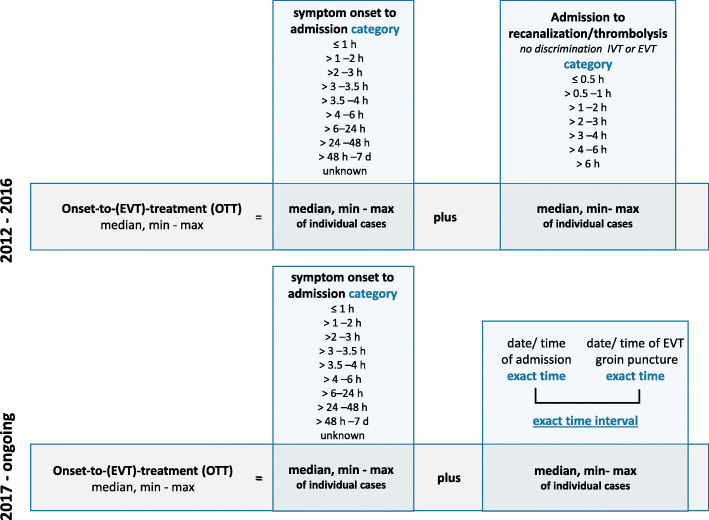
Derivation of the OTT estimate from the statewide stroke inpatient registry

We selected all patients with a discharge diagnosis of ischemic stroke (ICD-10 I63.x) with admission within 24 h from the time the patient was last seen well from 2012 to 2019 (*n* = 87.157) (Consort diagram in Supplement). To avoid duplicates, we included only cases with EVT at the documenting hospital and identified ‘drip-and-ship’ patients by ‘mode of admission’: ‘referral from other hospital’. We analyzed the discrepancy in OTTs of ‘drip-and-ship’ patients and ‘direct-to-center’ patients. In 2016, the registry was updated to include more detailed information on EVT. Therefore, we had to apply different selection strategies for the time period of 2012–2016 and the period from 2017 onwards (Fig. 4). To identify patients treated with EVT in the years 2012 to 2016, the indicators ‘intraarterial thrombolysis’ and ‘mechanical recanalization’ were used. Since 2017 these have been replaced by ‘intraarterial therapy’ specifically denoting EVT for acute stroke. Patients with an admission to EVT interval > 6 h were excluded in order not to analyze patients without primary EVT intention or with incorrect data entry. For the period 2012–2016, the OTT had to be approximated from the two variables ‘symptom onset to admission’ and ‘admission to recanalization’ both given in time strata of 30 and 60 min (Fig. 2). From 2017 onwards, the ‘admission to start of intra-arterial therapy’ interval could be calculated by the minute from two exact time stamps. We performed three calculations based on the minimum, median and maximum of each interval. Since we noted that in the cases from 2012 to 2016, the interval ‘admission to recanalization’ did not allow to discriminate between start of intravenous thrombolysis (IVT) and start of EVT, we excluded all ‘direct-to-center’ patients receiving IVT prior to EVT for the OTT analysis, reducing this cohort from *n* = 978 to *n* = 309 patients (Consort diagram in Supplement). From 2017 onwards, also ‘direct-to-center’ patients with IV thrombolysis could be included due to more specific time stamps.

For a closer characterization of the interhospital transfer workflow and DIDO time in patients being transferred from PSC to CSC, we performed a separate retrospective analysis collecting more detailed time stamps from a cohort of consecutive stroke patients referred for EVT from PSC to CSC in our regional stroke network INVN Rhine Main over a 3 months period (*n* = 37, response rate: 84.6%). The network consists of 8 PSCs and 6 CSCs and serves a population of approximately 3 million inhabitants in the metropolitan area of Frankfurt Rhine Main. We analyzed different workflow metrics: symptom onset to hospital admission, hospital admission to CT, CT to decision for EVT, EVT request to EVT commitment by CSC, transportation request to ambulance arrival and ambulance arrival to ambulance departure. We calculated the median and IQRs for those intervals and the total DIDO time interval. In order to identify specific problems causing delay in interhospital transfer workflow and their relative frequency, we asked for the main hurdles, giving several options to choose as well as a possibility to provide free text.

IBM SPSS Statistics 26.0 was used for statistical analysis.

## Results

### ‘Drip-and-ship’ patients are an important and growing proportion of EVT patients

**Fig. 3:**
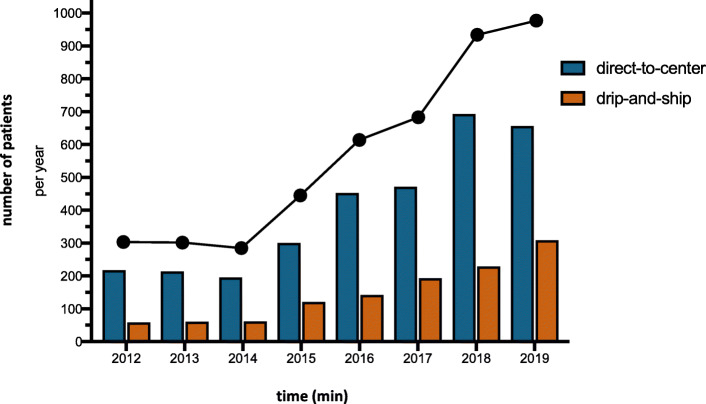
Proportion of drip-and-ship and direct-to-center among patients receiving EVT. The total number of patients receiving EVT throughout the years 2012 until 2019 in the federal state of Hesse, Germany is presented by the black dots and line graph. It shows an increase from n=287 in 2012 to *n*=984 in 2019. Patients directly admitted to a comprehensive stroke center with on-site mechanical thrombectomy service are referred to as ‘direct-to-center’, whereas patients first admitted to a primary stroke center and then transferred to a thrombectomy center are referred to as ‘drip-and-ship’

From 2012 to 2019, the annual numbers of EVT for stroke gradually increased from *n* = 253 (2.4% of all stroke patients admitted with time last seen well < 24 h) to *n* = 884 (7.5%) (Fig. 3). While ‘drip-and-ship’ patients represented 19.4% of all patients receiving EVT in 2012, their share rose to 31.3% in 2019.

### The time discrepancy of OTT between ‘drip-and-ship’ and ‘direct-to-center’ patients decreased over time

**Fig. 4:**
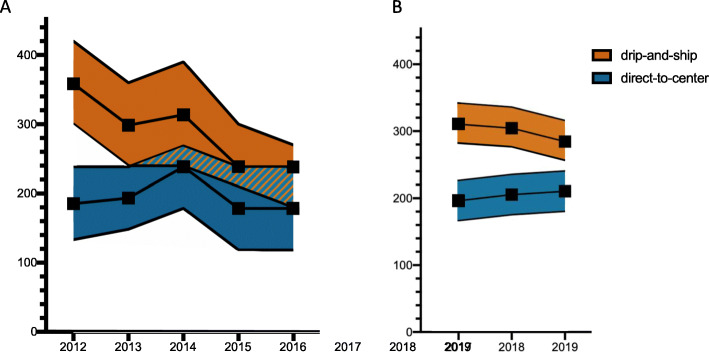
Onset to groin (OTG) time of drip-and-ship and direct-to-center patients. ‘Onset-to-groin’ (OTG) denotes the time interval from stroke symptom onset up to the time of groin puncture as the start of the endovascular procedure. The median onset to groin (OTG) times for the drip-and-ship and direct-to-center group were calculated from the BQS registry indicators ‘symptom onset to admission’ and ‘admission to initiation of therapy’ which were both recorded in strata of 30 or 60 min from 2012 to 2016 (**a**) whereas from 2017 onwards, a new registry indicator ‘admission to groin puncture’ derived from the actual times of patient arrival and groin puncture was added (**b**). To account for this approximation, we present the medians along with the shortest and the longest possible time intervals. The dotted line marks the change of the second indicator to a precise time in minutes with a subsequent artificial slight increase of median OTG in both groups

In 2012, stroke patients undergoing EVT after secondary transfer had a median OTT of 360 min whereas patients primarily treated at a CSC had a median OTT of 187 min (difference of medians: 173 min). This difference to the disadvantage of ‘drip-and-ship’ patients significantly declined to 60 min in 2016 with a median OTT in ‘drip-and-ship’ patients of 240 min and of 180 min in ‘direct-to-center’ patients (Fig. 4a). Analyzing the more precise OTT estimates from 2017 onwards, we found that onset-to-treatment times were actually longer but the OTT discrepancy between ‘direct-to-center’ and ‘drip-and-ship’ patients was in a similar range with a trend towards further improvements: differences of medians were 114 min in 2017, 99 min in 2018 and 74 min in 2019 (Fig. 4b).

### The most important delays of the door-in-door-out time (DIDO) occur after the decision for EVT

For a more granular appreciation of delaying factors, we collected process time stamps within the DIDO period in PSCs for all consecutive stroke patients transferred for EVT in the regional stroke network INVN Rhine Main. The total median DIDO time in the observed time period (January 2020–March 2020) was 92 min (IQR 69–110). The medians of the DIDO were 103 min in January, 89 min in February and 96 min in March, no meaningful trend could be detected in this short time period.

**Fig. 5:**
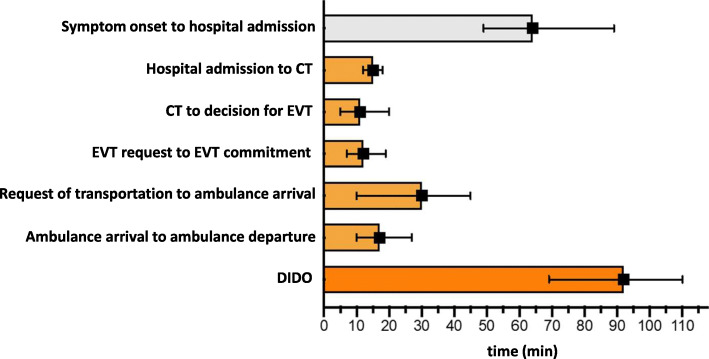
Time metrics of interhospital transfer. The time from admission to discharge from the primary hospital to the thrombectomy center is also referred to as DIDO (‘door-in-door-out’ (DIDO) time). Time metrics in this graph stem from a consecutive collection of time stamps of all patients transferred for EVT within the interdisciplinary neurovascular network Rhine-Main over a three months period

We observed that while the time-to-brain imaging interval met guideline recommendations with a median of 15 min and the decision for EVT was reached within a median time of 11 min (IQR 5–20) from brain imaging, the most important delays of the DIDO occurred after these initial steps (Fig. 5).

The PSCs usually have to make requests for EVT at a CSC by phone and due to local regulations can often only organize transportation if a CSC accepts the patient for EVT. In addition the interval from request of transportation for transfer and arrival of the ambulance at the PSC is frequently time-consuming.

Another time-consuming process was the preparation of the relaying emergency crew, especially due to medical measures that had to be initiated before departure to CSC. The most common causes for delay as judged by the PSCs are given in Table 1.

**Table 1:** Causes of delay in patient referral as reported by PSCs within the interdisciplinary neurovascular network Rhine-Main over a three months period

Causes of delay	Number of cases	%
**Lack of capacity in CSC, calls to several CSCs**	8	21.6
**Problems around decision for MT**	6	16.2
**Delay during transportation**	6	16.2
**Discussion with family members of the patient**	5	13.5
**Delay in teleradiological presentation**	5	13.5
**Lack of transportation capacity**	5	13.5
**Acute medical problems**	5	13.5
Problems with communication to CSC	3	8.1
Delay in first imaging	3	8.1
Incorrect admission diagnosis	1	2.7
Delay in LVO-detection	1	2.7
Error in internal workflow procedure	1	2.7
Difficulties obtainig medical history	1	2.7

### IVT prior to EVT did not lead to a prolongation of door-to-groin time (DTG)

It is sometimes stated that IVT may delay the initiation of EVT. Analyzing the cases since 2017 showed that prior IVT did not lead to a time delay on door-to-groin time (DTG) denoting the time period between arrival at the hospital and beginning of EVT (time of groin puncture). Patients in the direct-to-center group with prior IVT had a median DTG of 1:24 h in comparison to a median DTG of 1:28 h in patients with direct MT (*p* = 0.07), respectively. The years from 2012 until 2016 could not be evaluated because the data base did not distinguish between different recanalizing therapies (IVT or EVT) before 2017. A recent meta-analysis of 18 studies [13] comparing outcomes of stroke patients receiving EVT in a drip-and-ship vs. mothership paradigm found a significantly later initiation of IVT at PSCs in comparison of CSCs. We compared the door-to-needle times (DTN) of patients with presence of LVO who received IVT prior to transfer for EVT (drip-and-ship group) or EVT at the center (mothership group) and found no significant difference of the median DTN (35.0 min vs. 32.8 min, *p* = 0.12).

## Discussion

Our analysis of a virtually population-based quality assurance registry identifies ‘drip-and-ship’ patients as a relevant and increasing share of patients receiving EVT for acute stroke. Besides a general expansion of the indication for EVT, the greater awareness and readiness of PSCs to detect LVO and transfer patients for EVT is a significant contributor to the increment of EVT in the federal state of Hesse.

The increasing share of ‘drip-and-ship’ patients, which most notably occurred in parallel to an increase of CSCs regularly performing EVT, was accompanied by an impressive decrease of the OTT reducing the OTT discrepancy between ‘direct-to-center’ and ‘drip-and-ship’ patients to currently little more than 1 h. In parallel to the positive results from MR CLEAN announced in 2014 and the concordant results of the other thrombectomy trials, organizational milestones facilitating these improvements in OTT were initiated. Among these were the launch of a certification process for neurovascular networks by the German Stroke Society in 2013, a commitment from the ministry of health of our federal state to grant the highest priority to stroke patient PSC-CSC transports, a joint display of capacities in early 2016 as well as consented patient selection criteria and referral protocols by all 11 CSCs in the state from 2016 onwards.

Focusing on the DIDO we found that the basic emergency stroke procedures were already very well executed in PSCs. Most time-consuming aspects seem to arise after the decision for EVT and in the choice of CSC and transportation.

Analyzing the main barriers to a swift transfer identified by PSC stroke neurologists, we found that in more than 21.6% of transfer cases, it was necessary to contact several CSCs for a discussion of the patient case and confirmation of capacity. This was directly followed by problems around the decision to pursue EVT (such as high aged patients with several comorbidities, poor preliminary condition, long-standing symptom onset, etc.), named in 16.2% of transfer cases.

Some measures like an indicator of capacity and consented patient selection criteria (angiographic evidence of LVO, mRS ≤ 3, no extensive infarct/ASPECTS ≥5) have already been established to address these problems. Nevertheless, delays due to organization of EVT in the CSCs and the transportation are frequent. Problems with the teleradiological image transfer were also named in more than 10% of transfer cases. All of these argue for an easily-accessible joint platform to share imaging and thus facilitate the exchange between PSC and CSC.

Other frequent barriers were delays in transportation or a lack of transport capacity. These problems could benefit from a more direct involvement of paramedics and prehospital specialists. However, further improvement of these processes is urgently required. In order to achieve this goal the results of this analysis will be fed back to the hospitals, rescue coordination centers and preclinical service providers for further process improvement.

In contrast to current beliefs, IVT prior to EVT did not lead to a prolongation of DTG and we did not find longer DTN of patients receiving IVT at a PSC (drip-and-ship) in comparison to those thrombolyzed prior to EVT at a comprehensive stroke center (mothership) in Hesse in the observed period of time. This is an encouraging observation as prior IVT before EVT was shown to be associated with a better functional outcome [14].

The preliminary results of the prospective RACECAT trial presented at the ESOC 2020 show that PSCs may have their rightful place in acute stroke care if they follow streamlined procedures. The results of this trial argue against a premature implementation of preclinical triage and rerouting of patients with signs of severe stroke, at least in the trial’s regional context, and provides strong arguments for a further streamlining of the processes along the ‘drip-and-ship’ chain of acute stroke care. Most noteworthy, the discrepancy of the symptom onset to groin puncture time between ‘direct-to-center’ and ‘drip-and-ship’-patients in our cohort was quite similar to that observed in the RACECAT trial, providing solid evidence on the basis of real-world data that these metrics are not out of reach for other well-organized stroke networks around the world. Nevertheless, it must be considered that patient cohorts differed in both trials and also the geographical and infrastructural conditions cannot be transferred without restrictions.

One important factor in the equivalence of the ‘drip-and-ship’ and the “direct-to center” –concept in the RACECAT trial was the excellent performance of the PSCs. The DIDO achieved by PSCs in the RACECAT trial was little over 60 minutes [9] while the median DIDO achieved in our regional stroke network was 92 min. This reflects the potential of a further reduction of DIDO in our network with the focus on the identified time-consuming factors. As each one-hour delay in reperfusion by EVT is associated with a less favorable degree of disability and functional independence the discrepancy between ‘drip-and-ship’ and ‘direct-to-center’ patients should further be diminished [3].

Pondering the lessons that other stroke networks can learn from the RACECAT consortium, we think that the early identification of EVT candidates by paramedics in the field was one crucial factor. We assume that this spurred the paramedics’ awareness of a stroke patient’s eligibility for EVT and allowed them to prepare early on for an onward transport to a CSC in case of LVO detection. A second decisive element may have been the central neurologist on call who received the RACE scale score and a brief medical history from the paramedics and thereafter decided against randomization in almost every third case. This obviously added specificity allowing the PSCs to focus on the most likely EVT candidates.

Concerning the limitations of our dataset, a certain imprecision cannot be circumvented even after the databank workover of 2016 because the onset-to-admission time is given in time strata of 1 h. We used medians, minima and maxima of the individual patients’ onset-to-arrival times to derive our OTT estimates. The medians of these estimates are very similar to the RACECAT data (RACECAT: OTT for EVT of 270 min in ‘drip-and-ship’ patients vs. 214 min in ‘direct-to-center’ patients vs. Federal State of Hesse: 286 min vs. 212 min). And even the maxima of these estimates presenting the worst case scenario (316 min vs. 242 min) would not result in a significantly larger discrepancy in OTT between the two referral strategies. This is all the more reassuring as we included all patients into our analysis in contrast to the RACECAT trial excluding patients with an mRS > 2 or reduced life expectancy who often pose more difficult ethical questions concerning the prospective worth of the intervention.

## Conclusions

Our data from the quality assurance registry of federal state of Hesse show that the access to timely endovascular treatment has increased considerably for patients primarily admitted to a PSC. In parallel to this, we found an overproportioned increase in drip-and-ship patients reflecting the greater awareness and readiness of PSCs to detect LVO and refer patients for EVT. We observed a decreasing discrepancy of OTT for EVT between the two referral strategies. Currently, this discrepancy observed in our federal state, representative for stroke patients in Germany lies in the range of that observed in the RACECAT trial showing that the results of RACECAT should not be dismissed as “out of reach” for real world practice. By contrast, stroke networks should strive for an optimization of the drip-and-ship chain of acute stroke care and a DIDO of 60 min. Following our results optimizing DIDO is of immense importance, special attention must be paid to the EVT request at CSC and transportation process of the patients.

## Supplementary Information

**Additional file 1.** Consort diagrams for the time intervals 2012–2016 and 2017–2019.

## Data Availability

The datasets during and/or analysed during the current study available from the corresponding author on reasonable request.

## Abbreviations

IV-t-PA: Intravenous tissue plasminogen activator
AIS: Acute ischemic stroke
LVO: Large vessel occlusion
MT: Mechanical thrombectomy
AHA: American Heart Association
PCI: Percutaneous coronary intervention
PSC: Primary stroke center
CSC: Comprehensive stroke center
IVT: Intravenous thrombolysis
EVT: Endovascular therapy
DTG: Door-to-groin
OTG: Onset-to-groin
OTT: Onset-to-therapy
mRS: Modified rankin scale
CT: Computed tomography
NIHSS: National Institutes of Health Stroke Scale
DIDO: Door in Door out Time
