# Supplementary figures and images for: Quality assurance data for regional drip-and-ship strategies- gearing up the transfer process
Source: Neurol Res Pract. 2021 Aug 2;3:38. doi: 10.1186/s42466-021-00136-x (PMC8327429; doi:10.1186/s42466-021-00136-x)

Supplement  
Consort diagram

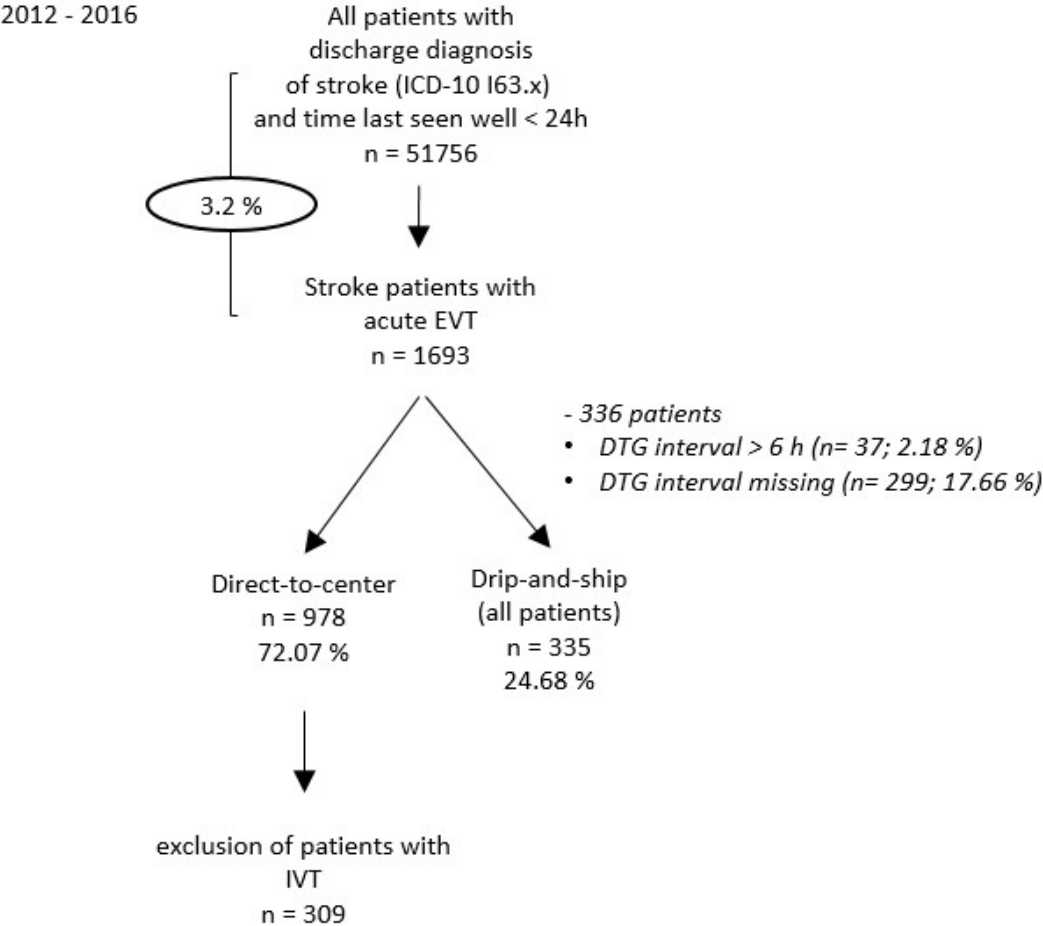

Supplement  
Consort diagram

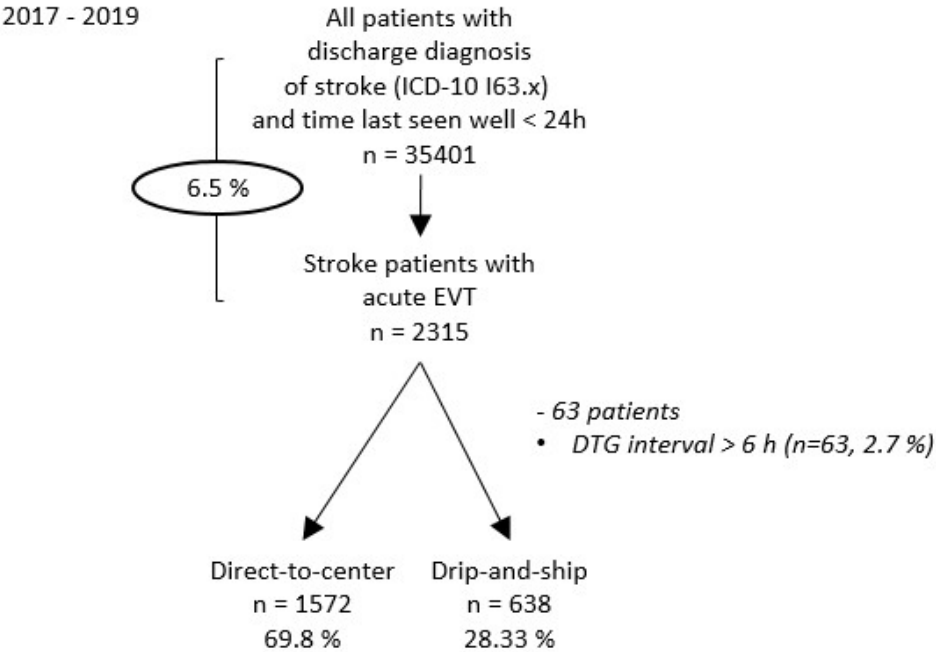

Supplement: Supplementary file 1 — Additional file 1. Consort diagrams for the time intervals 2012–2016 and 2017–2019. [file 42466_2021_136_MOESM1_ESM.pdf]
